# Establishment of a novel experimental model of infected anal fistula in rat

**DOI:** 10.1186/s42826-022-00125-4

**Published:** 2022-06-13

**Authors:** Meng Zhao, Aitong Wang, Leisheng Zhang, Hao Yu

**Affiliations:** 1 The Postdoctoral Research Station, School of Medicine, Nankai University, Tianjin, 300071 China; 2 National Engineering Research Center of Cell Products, AmCellGene Engineering Co., Ltd, Tianjin, 300457 China; 3 Tianjin Key Laboratory of Engineering Technologies for Cell Pharmaceutical, Tianjin, 300457 China; 4 Institute of Stem Cells, Health-Biotech (Tianjin) Stem Cell Research Institute Co., Ltd., Tianjin, 301700 China; 5 Key Laboratory of Radiation Technology and Biophysics, Institute of Biology & Hefei Institute of Physical Science, Chinese Academy of Sciences, Hefei, 230031 China; 6 Key Laboratory of Molecular Diagnostics and Precision Medicine for Surgical Oncology in Gansu Province & NHC Key Laboratory of Diagnosis and Therapy of Gastrointestinal Tumor, Gansu Provincial Hospital, Lanzhou, 730000 China

**Keywords:** Anal fistula modeling, DSS, BLV-SCEA, Rat model

## Abstract

Refractory Crohn's-like enterocutaneous fistula indicates the aggressive manifestation and lead to poor prognosis of patients. The development of multidisciplinary strategies for fistula administration largely subjects to the deficiency of animal model for disease remodeling and the underlying pathogenic mechanism. For the purpose, infected anal fistula model was conducted by BLV single-core electrolytic aluminum combined with dextran sodium sulfate. Notably, the inflammatory granulation tissue and inflammatory cell infiltration in the perianal tissue were arised on day 7 of the model by utilizing the Hematoxylin–eosin staining. With the aid of magnetic resonance imaging and signals of high-brightness. We intuitively observed the thickening and edema appeared in the fistula wall, which collectively suggested the formation of a fistula in the perianal area of the rat. Distinguish from the current models of anal fistula modeling including the body surface of fistula, backside of fistula and drainage wire of fistula, our model revealed multifaceted advantages such as quicker generation, higher modeling rate, preferable stability, better consistency, cost-effective, and in particular, more convenient to mimic clinical manifestations of anal fistula.

## Background

Anal fistula is a refractory and recurrent anorectal disease with a spectrum of complications such as purulent exudates, abscess, granulomatous inflammation, highly exposed fistula and intolerable itching [1, 2]. Currently, a series of surgical methods were introduced for the management of anal fistulas worldwide including incision/drainage, fistula removal, thread hanging, fistula tamponade and so on. However, these strategies usually showed remarkable shortcomings such as high rate of relapse, secondary infections and anal incontinence. To date, the deficiency of "standard surgical procedures" for anal fistulas patients as well as the objective evaluation system during surgery largely hinder the development of more effective therapeutic remedies in clinical practice. Notably, Colorectal Surgery Guidelines of the United States have proposed the condition of "survival along with fistula" for diagnosis and treatment of anal fistula [3–5]. Accordingly, it’s necessary to construct better anal fistula animal models to mimic the symptoms of anal fistula and dissect the similarities and differences among the different surgical methods based on the post-operative anal function, which plays an important role for the follow-up of effective administration of the disease.

As mentioned above, despite the reported methodologies (e.g., body surface, backside or canal drainage line) for constructing disease models by investigators in the field, yet most of the experimental models for more accurately mimicking the symptoms and pathological changes of anal fistula disease are largely obscure. In details, the majority of the current models are difficult to achieve the uniformity epithelialization of fistula and dissimilar from the formation of anal fistula in clinical practice [6–8].

## Main text

**Fig. 1:**
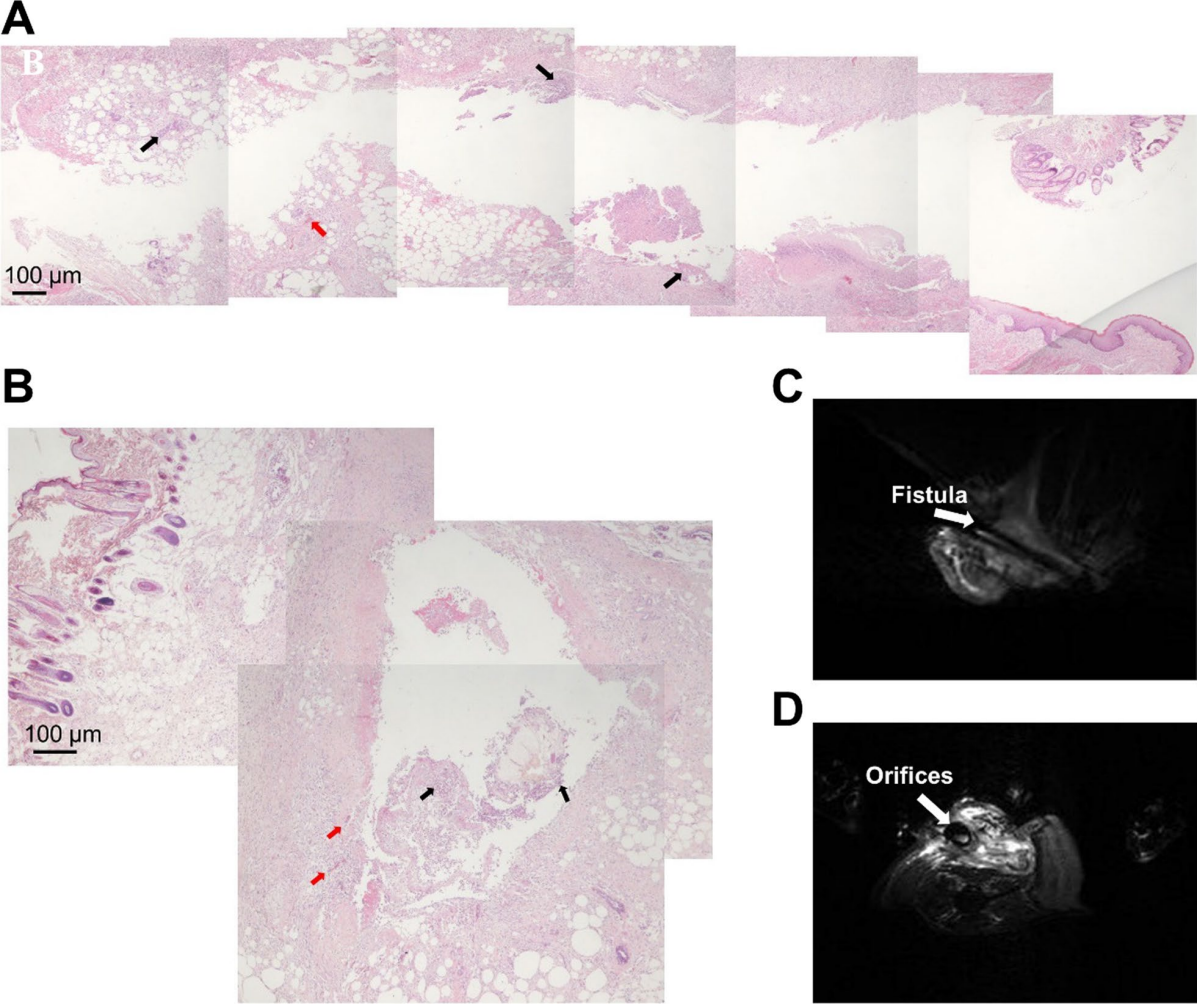
The inter-structure of the fistulous tract area after modeling. **A**–**B.** Representative images of histopathological examination by H&E staining according to the longitudinal section (**A**) and cross section of anal fistula (**B**). The red arrow and black arrow indicate the eosinophils and lymphocytes, respectively. Scale bar = 100 μm. **C**–**D.** T1 and T2-weighted morphological images of anal fistula based on BRUKER 9.4 T micro-MRI system for small animals. In details, the fistula (**C**) and orifices (**D**) were indicated by white arrow

In this study, we constructed an infection rat anal fistula model through BLV single-core electrolytic aluminum combined with dextran sodium sulfate (DSS). Firstly, we utilized an electric razor to shave the animal's hair and to remove the remaining of hair with a depilatory cream in the operation area after anesthesia administration. Secondly, leave the rats on the operating table in prone position and fix the limbs with tape. Using of the PVC-wrapped 2.5 mm^2^ aluminum wire to pass the anal sphincter with 1 cm from left or right side of the anus, followed by rotating and fixing rats with a suitable length after penetrating the skin to keep rats hanging around the anal sphincter. Thirdly, the rats were continuously given fresh water with 5% DSS addition for one week to generate the general symptoms of anal fistula including the increased inflammatory response, the red and swollen fistula, tissue fluid exudation, anus with locally moist, and fecal secretions. Fourthly, the pathological changes of anal fistula in rats were evaluated by routine sectioning and hematoxylin and eosin (H and E) staining. As shown by the histopathological examination, typical inflammatory granulation tissues in the fistula wall along with tissue destruction of mucous epithelium were intuitively observed. According to the longitudinal section and cross section of anal fistula, we also noticed substantial inflammatory cell infiltration such as lymphocytes, eosinophils and neutrophils in the mucosal layer of anal fistula, together with the red-stained necrotic tissues in the lacuna (Fig. 1A–B). Meanwhile, the formation of fistula in crissum along with the higher signal intensity of tissue around the fistula by T2 and T1-weighted morphological images were shown by MRI images (Fig. 1C–D). For instance, the phenomena of thickening and edema were institutively observed in the fistula wall, which effectively mimicked the clinical manifestations of anal fistula.

## Conclusions

Despite the continuous improvement in the remedies of anal fistula with various complications, yet a certain number of patients still endure the long-lasting torments both physically and mentally due to the deficiency of effective models for disease remodeling. For instance, the surface fistula model or the dorsal fistula model reveals no correlation with anal fistula, while the anal drainage fistula model has inherent defects in the consistency of fistula epithelialization and drainage biomaterials as well as the time-consuming process. Similarly, the anal screw wire fistula model has been reported with a modeling time for over 30 days but with low-efficient infectious corrosion in perianal region and the difficulty in removing the threaded wire.

Distinguish from the current animal models of "irrational" anal fistula, our model possessed three prominent advantages. Firstly, it preferably mimicked the symptoms of anal fistula patients with contaminated fistula wounds by feces on the occurrence of diseases in the clinic, which supplied overwhelming new model for dissecting the pathology and therapeutics. In particular, the results of MRI and H&E examinations were highly consistent with the signal intensity and pathological characteristics of anal fistulas. Secondly, this strategy with a higher success rate, a shorter molding cycle, more reliable repeatability, together with minimal surgical risks and cost-effective. In details, our rat anal fistula model was constructed by combining BLV single-core electrolytic aluminum with 5% DSS for one week, and all of the rats revealed typical pathomorphological (e.g., local wetting of anus, fecal secretions, purulent fistula) and histopathological (e.g., inflammatory granulation tissue, inflammatory cell infiltration) characteristics of anal fistula, which were distinguish from the current models with the multifaceted shortcomings as mentioned above. Thirdly, our model was adequate to imitate multiple complications of infectious anal fistula involved in Crohn's disease and ulcerative colitis. Collectively, our methodology would provide a novel "rational" model for further illuminating the pathogenesis and drug development for anal fistula-associated intractable and recurrent disorders in clinical practice in future.

## Data Availability

All data generated or analyzed during this study are included in this published article. Meanwhile, the datasets used and analyzed during the current study are also available from the corresponding author on reasonable request.

## Abbreviations

BLV-SCEA: BLV single-core electrolytic aluminum
DSS: Dextran sodium sulfate
PVC: Polyvinyl chloride
MRI: Magnetic resonance imaging
